# Effect of p53 on pancreatic cancer-glucose tolerance abnormalities by regulating transglutaminase 2 in resistance to glucose metabolic stress

**DOI:** 10.18632/oncotarget.19402

**Published:** 2017-07-19

**Authors:** Xiao Su, Xiangyi He, Qiwen Ben, Weiyi Wang, Huan Song, Qiao Ye, Yi Zang, Weiguang Li, Ping Chen, Weiyan Yao, Yaozong Yuan

**Affiliations:** ^1^ Department of Gastroenterology, Ruijin Hospital, Shanghai Jiaotong University School of Medicine, Shanghai 200025, China; ^2^ Department of Gastroenterology, Eastern Hepatobiliary Hospital, Second Military Medical University, Shanghai 200433, China

**Keywords:** transglutaminase2, p53 gene, glucose metabolic stress, diabetes-associated pancreatic cancer

## Abstract

Pancreatic ductal adenocarcinoma (PanCa) is an extremely lethal disease characterized by mutations of p53 in up to 70% of cases. Our previous studies have confirmed that hyperglycemia may be the first clinical manifestation for the early diagnosis of PanCa. In this article, we showed that targeted knockdown of TG2 or p53 in tumor cells led to decreased cell survival in response to glucose deprivation, while this phenomenon was abolished by combined inhibition of TG2 and p53. We observed that inhibition of TG2 or p53 sensitized glucose deprivation resistance through an intracellular reactive oxygen species (ROS) pathway and the induction of Bcl-2. Moreover, to understand whether pancreatic cancer cells with TG2 and p53 combined interference had possible effects on pancreatic β cells, we performed studies comparing pancreatic cancer cells with TG2 and p53 combined interference and pancreatic β cells. We discovered that the supernatant of pancreatic cancer cells withTG2 and p53 combined interference decreased cell survival in pancreatic β cells. Following the creation of an orthotopic pancreatic cancer mouse model, we revealed glucose tolerance abnormalities in the pancreatic cancer mouse model with TG2 and p53 combined interference, indicating a possible mechanism for damage of βcells in pancreatic cancer. Taken together, our findings establish roles for TG2 and p53 in response to glucose deprivation in pancreatic cancer cells. The relationship between TG2 and p53 suggests a possible mechanism for glucose tolerance abnormalities-associated pancreatic cancer and could have therapeutic potential for cancer treatment and diagnosis.

## INTRODUCTION

Pancreatic cancer is characterized by poor early diagnosis, high mortality rate, and low overall survival (no more than 5% at 5 years). [1-4] New onset diabetes, especially occurring within six months, increases with the degree of malignancy of pancreatic cancer and decreases after treatment[5, 6]. In a published clinical investigation, our group confirmed that 65% of patients with diabetes can be cured by surgical resection of pancreatic cancer[7-9]. However, the mechanism by which hyperglycemia develops in patients with pancreatic cancer has not been elucidated[5, 6]. The pancreatic cancer cell microenvironment is thought to be strictly selected and the metabolic stress resistance in pancreatic cancer cells is thought to depend on a variety of complex mechanisms[10]. Therefore, in this present study, we chose to investigate the mechanism of hyperglycemia that develops in pancreatic cancer and is dependent upon metabolic stress.

Tissue transglutaminase2 (TG2) is a member of the transglutaminase family and catalyzes calcium-dependent, post-translational modification of proteins[11, 12]. TG2 is implicated as being ubiquitously expressed in cancer cells and is involved in the regulation of several physiological processes such as cell death, cell adhesion and differentiation, survival, and cell migration. [13-15] Some evidence has clarified that TG2 plays a crucial role in hypoxia-induced stress in pancreatic cancer[16-18]. However, the biological role of TG2 in glucose deprivation stress is not clearly defined. Although TG2 is mainly a cytosolic protein, it can translocate to the nucleus with pRb, p53, and histone combined regulation of cell-specific functions[19-21]. Indeed, the p53 tumor suppressor gene has been proposed as a key determinant in metabolic stress resistance in pancreatic cancer cells [22-24] However, the identification of novel targets of metabolic stress resistance is urgently needed[10]. Microenvironmental stress in pancreatic cancer may be the causative mechanism of hyperglycemia in patients with pancreatic cancer[25, 28]. Therefore, study of the key molecules regulating stress to the pancreatic cancer cell microenvironment would enable better understanding of early pancreatic cancer occurrence and development.

In the present study, our results demonstrated that higher expression and activation of TG2 played a decisive role in glucose stress resistance in pancreatic cancer cells. Interestingly, the sensitization effects were abolished by inhibition of p53. Moreover, we clarified that the sensitization effects to glucose stress of TG2 and p53 were attributed to the induction of oxidative stress and Bcl-2 expression. Furthermore, our results discovered that the supernatant of pancreatic cancer cells with TG2 and p53 combined interference decreased cell survival in pancreatic β cells. Of note, our results demonstrated glucose tolerance abnormalities in an orthotopic pancreatic cancer mouse model with TG2 and p53 combined interference, indicating a possible mechanism for hyperglycemia occurring in pancreatic cancer.

## RESULTS

### Tolerance to extreme glucose deprivation in pancreatic cancer cell lines with different expression of TG2 and p53

**Figure 1:**
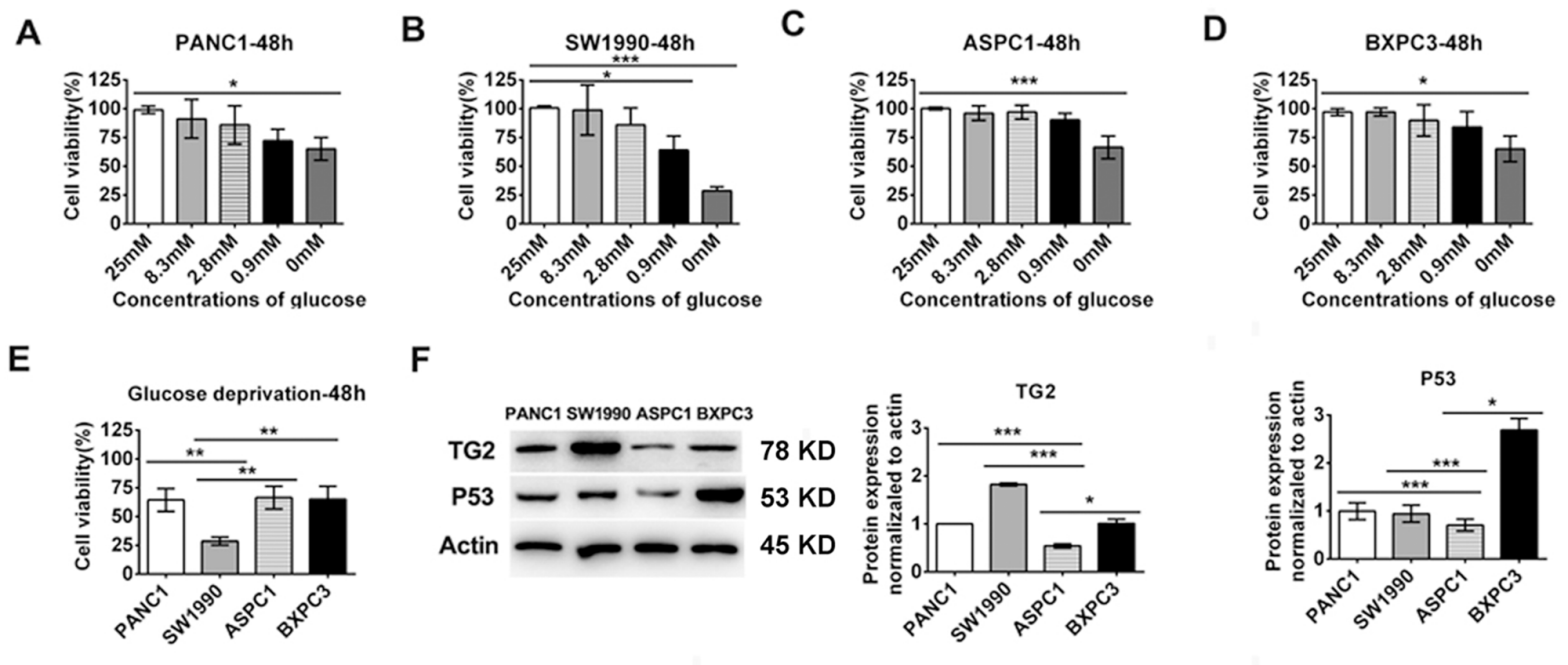
The cell lines displayed differential expression of TG2 and p53 and sensitivities to glucose starvation **(A-D)** The cell viability of PANC1, ASPC1, SW1990, BXPC3 cells treated with a series concentrations of glucose deprived cultural medium for 48 h. **(E)** The differential cell viability to glucose starvation in the four cell lines at 48h. **(F)** Western blot analysis of the expression of TG2 and p53 in pancreatic cancer cells (PANC1, SW1990, ASPC1 and BXPC3). The protein expression normalized to actin was analyzed. Data are expressed as mean ± SD (n = 3). *, p <0.05; **, p < 0.01; * *, p < 0.001.

In order to investigate tolerance to extreme glucose deprivation in various pancreatic cells, we first examined the cell viability of PANC1, ASPC1, SW1990, and BXPC3 cells treated with a series of concentrations of glucose-deprived culture medium for 2 or 3 days. As shown in Figure 1A-D, we found that the cell lines displayed differential sensitivities to glucose starvation. PANC1, ASPC1, and BXPC3 cells were more resistant than SW1990 cells to glucose stress after 48 h (Figure 1E). All the tested cells displayed resistance to extremely low glucose stress (Figure 1A-D). Similar results were obtained after 72 h (Supplementary Figure 1). As TG2 combine with p53 can regulation of cell-specific functions for significance,[19, 21] we compared the total protein expression levels of TG2 and p53 in the pancreatic cell lines using western blot analysis. As shown in Figure 1F, the total protein expression levels of p53 in BXPC3, SW1990 and PANC1 cells were higher than in ASPC1 cells. The total protein expression levels of TG2 in SW1990 cells were higher than in the other cells (Figure 1F). Moreover, the total protein expression levels of TG2 and p53 in ASPC1 cells were lowest compared to the other control levels (Figure 1F). These data suggest that the function of TG2 and p53 in extreme glucose stress in pancreatic cells need further study.

### TG2 up-regulation and activation is required for resistance to glucose starvation stress

**Figure 2:**
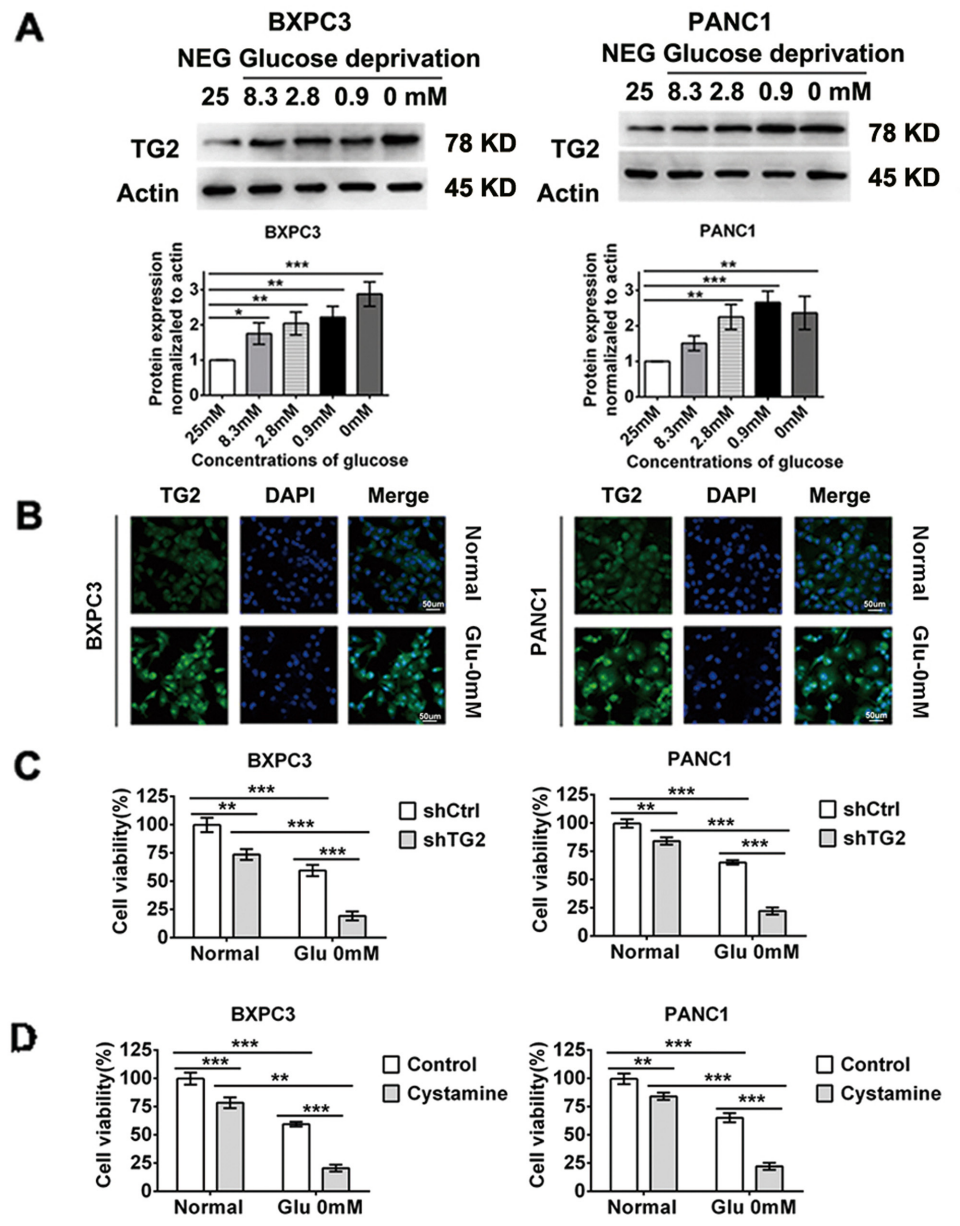
TG2 up-regulation and activation is required in resistant to glucose starvation stress **(A)** Western blot evaluated the TG2 expression changes in conditions of decreased concentration of glucose. The protein expression normalized to actin was analyzed. **(B)** Confocal performed to measure TG2 enzymatic activities in conditions of glucose starvation. **(C)** BXPC3 and PANC1 cells expressing shCtrl or shTG2 were assayed for their ability to in resistant to glucose starvation stress. **(D)** The cell viability were measured using TG2 specific inhibitors in BXPC3 and PANC1 cells under glucose starvation. Data are expressed as mean ± SD (n = 3). *, p <0.05; **, p < 0.01; * *, p < 0.001.

To investigate whether TG2 protein responds to glucose stress in pancreatic cancer cells, we evaluated the changes in TG2 expression and activity in conditions of glucose starvation. As shown in Figure 2A, increased levels of TG2 were correlated with decreased concentrations of glucose as estimated by western blot analysis. The TG2 protein level increased after 48 h of glucose starvation. In situ enzymatic activity assays to measure TG2 activity showed that glucose starvation induced elevated TG2 activity over control levels after 48 h of glucose starvation (Figure 2B). In an effort to identify TG2 regulation, we attempted to determine the sensitivities of BXPC3 and PANC1 cells treated with glucose deprivation in which TG2 expression was blocked by shRNA interference and specific inhibitors. TG2 expression was examined by western blot analysis after shRNA transfection (Supplementary Figure 2). As shown in Figure 2C, knockdown of TG2 markedly decreased viability in BXPC3 and PANC1 cells treated with glucose starvation for 48 h. Similar results were obtained using TG2 specific inhibitors in BXPC3 and PANC1 cells (Figure 2D). Cell viability with a series of concentrations of glucose was demonstrated in BXPC3 and PANC1 cells (Supplementary Figure 3). These data indicate that TG2 induces resistance to glucose stress in pancreatic cancer cells. In order to determine the regulation of p53 on TG2, we investigate p53 accumulation in response to glucose starvation next.

### Role of p53 in regulating TG2 up-regulation in resistance to glucose starvation stress

**Figure 3:**
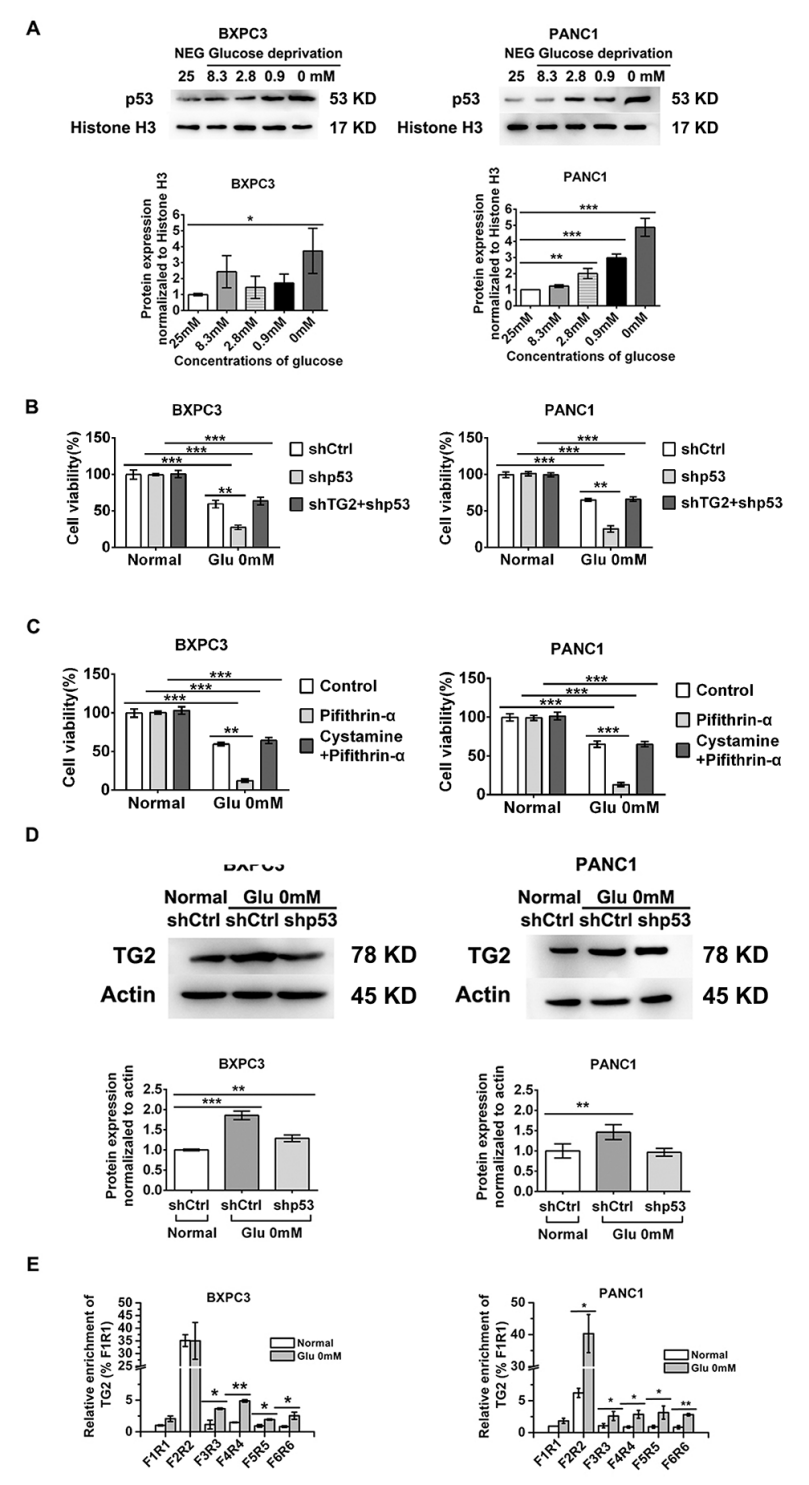
Role of p53 nuclear activation in regulating TG2 up-regulation in resistant to glucose starvation stress **(A)** Western blot evaluated the p53 nuclear expression changes in conditions of decreased concentration of glucose. The protein expression normalized to Histone 3H was analyzed. **(B)** BXPC3 and PANC1 cells expressing shCtrl, shTG2or shTG2+shp53 were assayed for their ability to in resistant to glucose starvation stress. **(C)** The cell viability was measured using p53 and TG2 specific inhibitors in BXPC3 and PANC1 cells under glucose starvation. **(D)** After p53 was knocked down, the TG2 expression was evaluated by western blot under glucose starvation. The protein expression normalized to actin was analyzed. **(E)** Different affinity of p53 binds to TG2 was evaluated by chip using specific primer pairs. Data are expressed as mean ± SD (n = 3). *, p <0.05; **, p < 0.01; * *, p < 0.001.

Since p53 accumulation is known to occur in cellular stress [23, 27], we asked whether p53 was induced in conditions of glucose starvation in pancreatic cancer cells. Consistently, we found that increased nuclear accumulation of p53 was correlated with decreased concentrations of glucose (Figure 3A). Knockdown of p53 by shRNA transfection markedly decreased the viability of BXPC3 and PANC1 cells treated with glucose starvation (Figure 3B). The p53 expression was inhibited by approximately 90% with p53 shRNA on western blot analysis (Supplementary Figure 2). Moreover, inhibition of p53 by specific inhibitors markedly decreased the viability of BXPC3 and PANC1 cells treated with glucose starvation (Figure 3C). This maybe because that p53 loss determine increased glucose uptake during PDAC progression,[29] so knockdown of p53 by shRNA transfection increased the sensitivity of PDAC cells treated with glucose starvation (Figure 3B). Surprisingly, combined knockdown of TG2 and p53 did not significantly influence cell survival in response to glucose starvation stress in BXPC3 and PANC1 cells (Figure 3B). Similar results were obtained using p53 specific inhibitors (Figure 3C). Cell viability with a series of concentrations of glucose was demonstrated in BXPC3 and PANC1 cells (Supplementary Figure 4). These findings suggest that p53 regulates TG2 in resistance to glucose metabolic stress.

Because up-regulation of TG2 was induced by glucose starvation, we sought to determine the regulation of p53 on TG2 in response to glucose starvation. Western blot analysis demonstrated that up-regulation of TG2 was reduced after inhibition by p53 shRNA in BXPC3 and PANC1 cells (Figure 3D). While expression of TG2 was still increased after inhibition by p53 shRNA in BXPC3 cells, this indicated that a portion of up-regulation of TG2 may be affected by p53 inhibition (Figure 3D), while this suggests that up-regulation of TG2 was not be regulated by p53 (Figure 3D). To determine whether nuclear p53 induced effects on TG2 expression, we used chromatin immunoprecipitation (ChIP) to investigate specific primer pairs mediated via direct binding effects on promoter activity to search for TG2 response elements. Our results demonstrated that under glucose starvation stress conditions, p53 binds to TG2 with highest affinity within a region located 3600–3800 bp of the transcription start site in BXPC3 cells (F4R4; Figure 3E), while under glucose starvation stress conditions, p53 binds to TG2 with highest affinity within a region located 700–900 bp of the transcription start site in PANC1 cells (F2R2; Figure 3E). Furthermore, p53 binds to TG2 with higher affinity within a region located 2000–2200 bp, 5000–5200 bp, and 5900–6100 bp of the transcription start site in BXPC3 and PANC1 cells under glucose starvation stress conditions (F3R3, F5R5, F6R6; Figure 3E). All these results clarified that nuclear p53 could bind to TG2 by specific transcription start sites in resistance to glucose starvation stress. These findings suggest that combined inhibition of TG2 and p53 may induce a specific microenvironment for pancreatic cancer cells.

### Inhibition of p53 and TG2 increased ROS and Bcl-2 production under glucose starvation conditions

**Figure 4:**
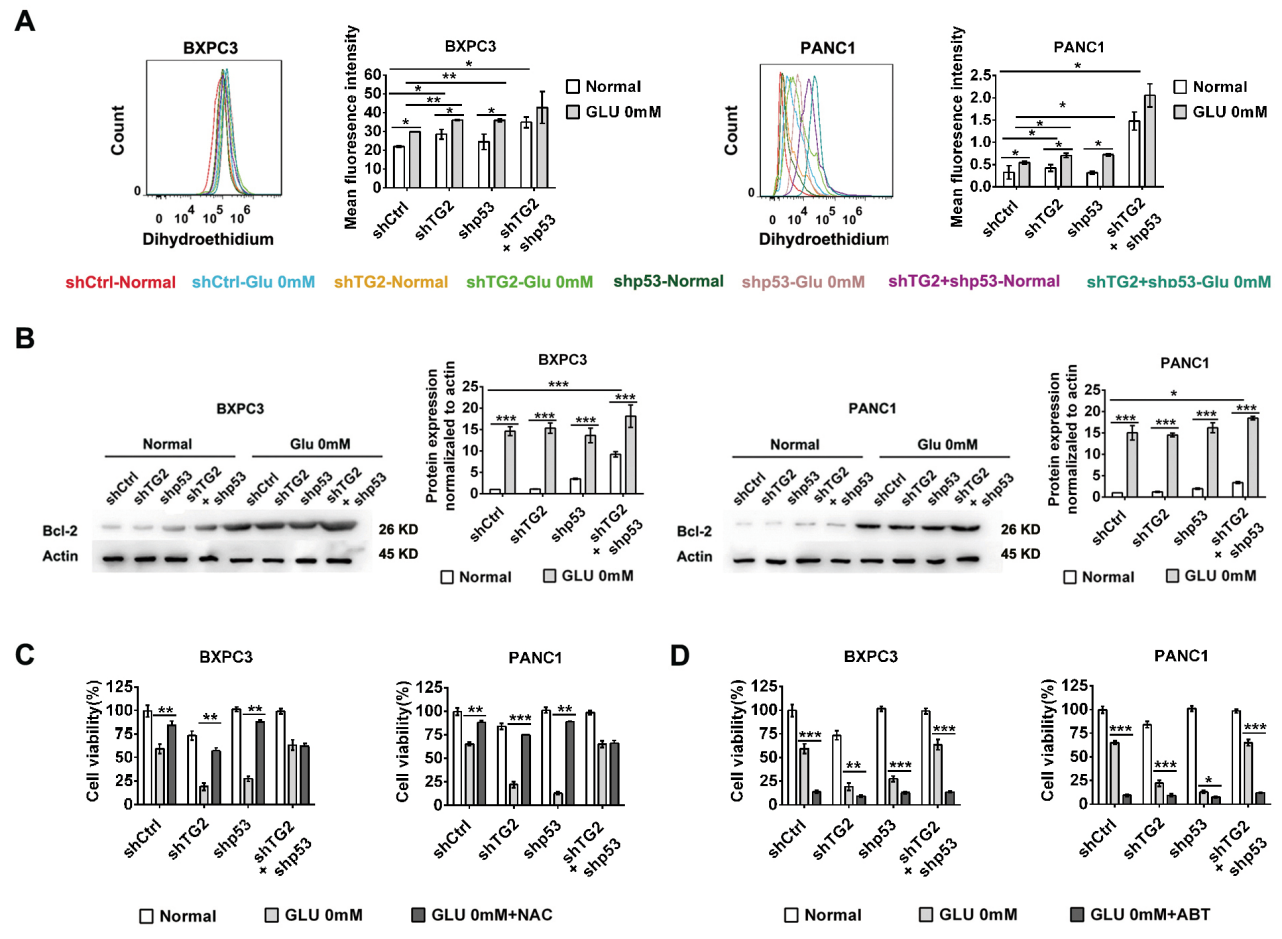
Intracellular reactive oxygen species (ROS) and Bcl-2 expression were measured in resistant to glucose starvation stress under regulation of p53 and TG2 **(A)** The production of intracellular reactive oxygen species (ROS) were measured by the flow cytometry in BXPC3 and PANC1 cells expressing shCtrl, shTG2, shp53 or shTG2+shp53 under normal and glucose starvation. The histogram (left part) and mean fluorescence intensity of the cells (right part) were shown. **(B)** Western blot evaluated the Bcl-2 expression changes in BXPC3 and PANC1 cells expressing shCtrl, shTG2, shp53 or shTG2+shp53 under normal and glucose starvation. The protein expression normalized to actin was analyzed. **(C)** The cell viability were measured using NAC in BXPC3 and PANC1 cells under glucose starvation. **(D)** The cell viability were measured using Bcl-2 specific inhibitors in BXPC3 and PANC1 cells under glucose starvation. Data are expressed as mean ± SD (n = 3). *, p <0.05; **, p < 0.01; * *, p < 0.001.

To determine whether oxidative stress and anti-apoptosis were involved in resistance to glucose starvation stress under the regulation of p53 and TG2, the production of intracellular reactive oxygen species (ROS) and Bcl-2 expression were measured[24, 26]. Our results demonstrated that higher ROS activity was generated under glucose starvation stress compared to basal conditions in control, shTG2, and shp53 cells except shTG2+shp53 cells (Figure 4A). Moreover, the highest ROS production was acquired in BXPC3-shTG2+shp53 cells when compared to control, shTG2, and shp53 cells, suggesting that higher ROS production was increased after shTG2 and shp53 combined interference (Figure 4A). The higher ROS production was acquired in BXPC3-shTG2 cells when compared to control cells, suggesting that higher ROS production was increased after shTG2 interference (Figure 4A). Total ROS levels were higher in shTG2 and shp53 cells than control under glucose starvation stress (Figure 4A). Similar results were obtained in PANC1 cells (Figure 4A). Considering Bcl-2 complexes are common targets for activating anti-apoptotic family proteins, we then measured Bcl-2 expression in different cells under glucose starvation stress. The results demonstrated that Bcl-2 expression levels were increased under glucose starvation stress compared to basal conditions (Figure 4B). Importantly, Bcl-2 expression levels were increased in BXPC3-shTG2+shp53 cells compared to control, shTG2, and shp53 cells (Figure 4B). Similar results were obtained in PANC1 cells (Figure 4B). Next, we treated cells with N-acetyl-L-cysteine (NAC), which is an ROS scavenger, under glucose starvation stress. The results indicated that control, shTG2, and shp53 cells underwent glucose starvation stress after treatment with NAC (Figure 4C), whereas ROS inhibition did not significantly influence cell survival in BXPC3-shTG2+shp53 cells in response to glucose starvation stress (Figure 4C). Similar results were obtained in PANC1 cells (Figure 4C). After treatment of cells with ABT, which is a specific inhibitor of Bcl-2, the resistance to glucose starvation was depressed in BXPC3-shTG2+shp53 cells (Figure 4D). Similar results were obtained in PANC1 cells (Figure 4D). Altogether, these data suggest that ROS production is required for p53- and TG2-induced resistance under glucose starvation stress. However, increased ROS production was not the cause for resistance to glucose starvation stress in shTG2+shp53 cells and increased expression of Bcl-2 may be responsible for resistance to glucose starvation stress in these cells. These results couldn’t provide direct evidences for the role of p53 or TG2 for ROS, anti-apoptotic Bcl-2 production under glucose starvation. While p53 or TG2 might regulate other molecules or pathway to induce ROS, anti-apoptotic Bcl-2 production under glucose starvation.

### Pancreatic cancer cells with silenced p53 and TG2 combination produced depressed survival of pancreatic β cells

**Figure 5:**
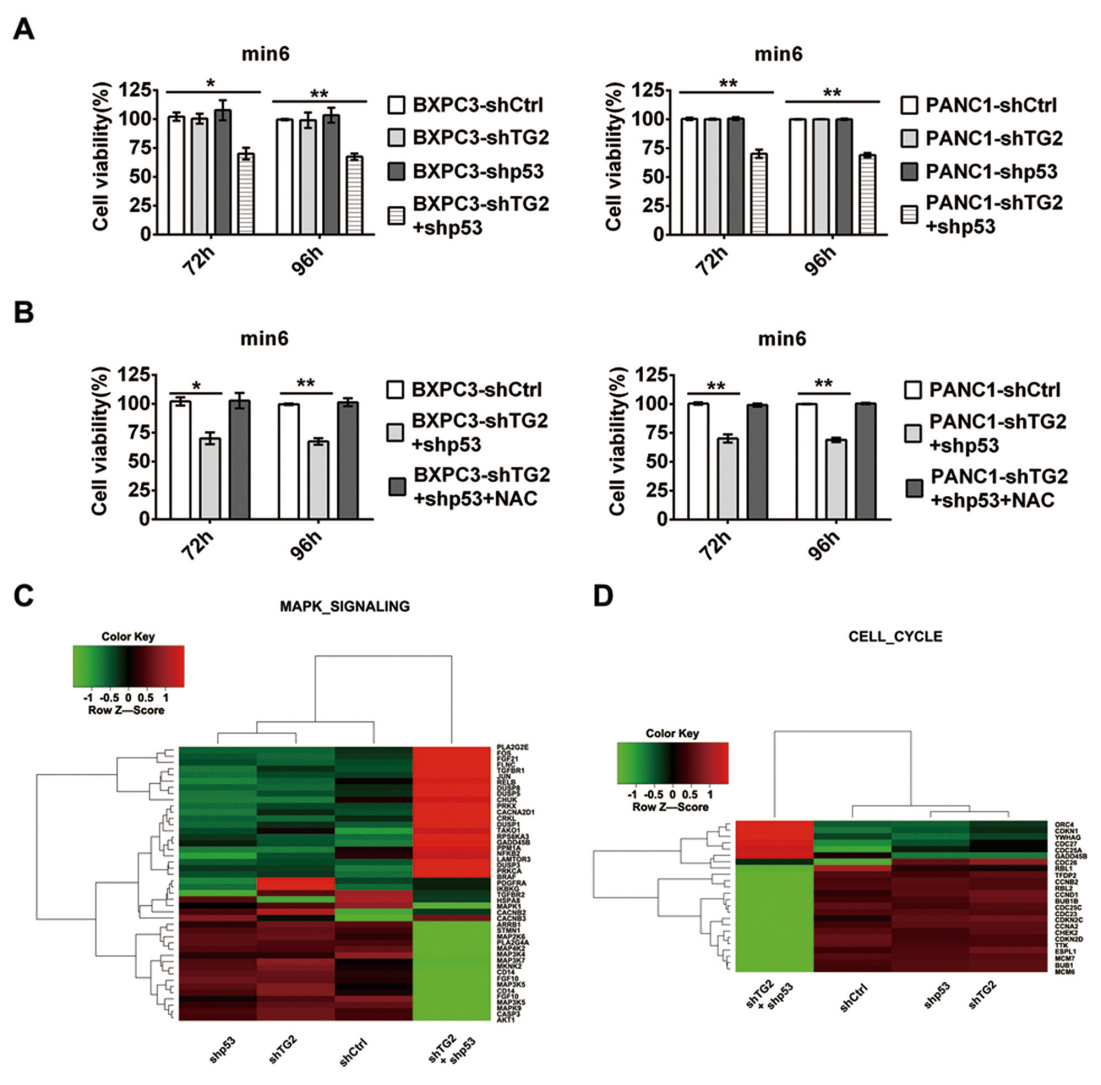
Pancreatic cancer cells with silenced TG2 and p53 combinations affect the survival of pancreatic β cell **(A)** The supernatant of pancreatic cancer cells expressing shCtrl, shTG2, shp53 or shTG2+shp53 treated pancreatic β cell min6. The cell survival in min6 were measured after 72h, 96h treatment. **(B)** Pretreatment of NAC in min6, the cell survival in min6 were measured after the supernatant of pancreatic cancer cells treatment at 72h,96h. **(C) (D)** Microarray experiments were performed in pancreatic β cells after treatment with the supernatant of different pancreatic cancer cells. Heat map of genes in MAPK signaling pathway in min6 cell demonstrated after treated by the supernatant of shCtrl, shTG2, shp53, shTG2+shp53 transfeced BXPC3 cell (C). The other interesting cluster, Cell cycle were demonstrated (D). Data are expressed as mean ± SD (n = 3). *, p <0.05; **, p < 0.01; * *, p < 0.001.

According to the above results, the higher ROS production was acquired in shTG2+shp53 cells and shTG2 when compared to control cells. As oxidative stress and products was responsible for damage in β cells and diabetes,[10] so we considered that silenced TG2 and p53 combination may provide a specific microenvironment in pancreatic cancer cells. Next, we analyzed whether silenced TG2 and p53 combination in pancreatic cancer cells induced effects on adjacent pancreatic β cells. We found that the supernatant of BXPC3-shTG2+shp53 cells decreased cell survival by approximately 30% in the min-6 cell after 72 h and 96 h of treatment (Figure 5A). The cell survival was not changed in the min-6 cell after 24 h and 48 h of treatment (Supplementary Figure 5). Consistent data were obtained in PANC1 cells (Figure 5A). Thereafter, we investigated whether ROS play a role in these effects. Interestingly, the depression disappeared after treatment with NAC (Figure 5B), while the total ROS in the supernatant did not show any significant difference between all cell types (data not shown). These results indicate that silenced p53 and TG2 combination in pancreatic cancer cells may provide a specific microenvironment for adjacent β cells to reduce their survival. Interestingly, inhibition of ROS plays a protective role for β cells and ROS in the supernatant were not responsible for the damage. Microarray experiments were performed to assess further which pathway is the critical regulator in the min-6 cells after treatment with the pancreatic cancer cell supernatant. The set included many genes involved in the MAPK and Cell cycle signaling pathways, which are the most significant (Figure 5C,5D). We calculated change in expression for each gene affected by the supernatant treatment. The expression of genes in the MAPK signaling pathway significantly changed (Figure 5C). Furthermore, the expression of genes in the Cell cycle signaling pathway was significantly affected (Figure 5D).

### Pancreatic cancer cells with silenced p53 and TG2 combination reduced glucose tolerance in an orthotopic mouse model

**Figure 6:**
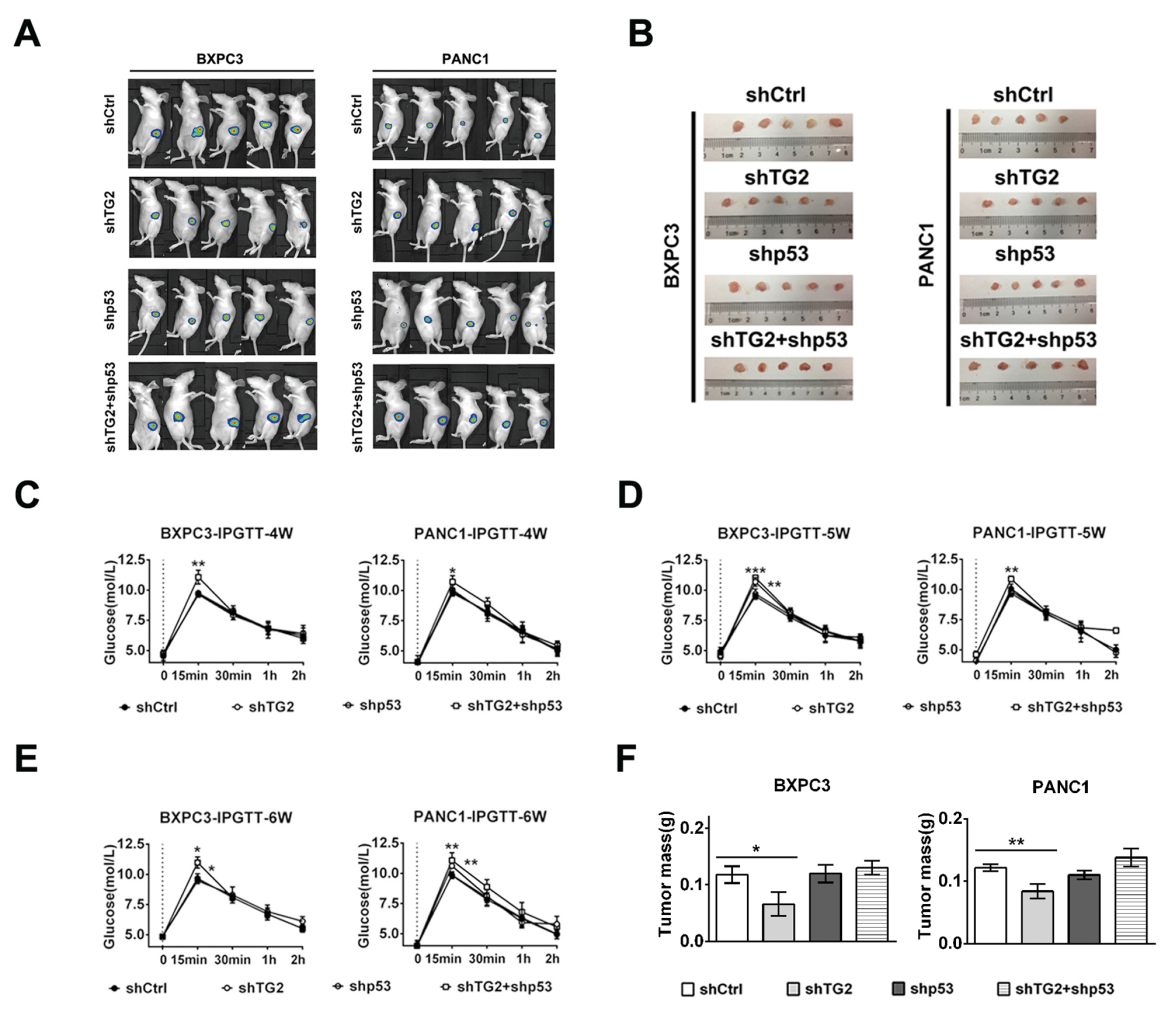
Pancreatic cancer cells with silenced TG2 or p53 combinations reduced the glucose tolerance in orthotopic pancreatic mice model After mice were randomly assigned to 4 groups: shCtrl, shTG2, shp53 or shTG2+shp53, the orthotopic pancreatic mice model was successfully established for 5 mice per group. **(A)** The tumor luminescent images taken after 4 weeks. **(B)** Gross morphology of orthotopic pancreatic cancer. **(C) (D) (E)** Glucose tolerance test in the 4 groups mice at 4^th^, 5^th^, 6^th^ week. **(F)** The excised tumors were weighted at the end point. The tumor weight of the 4 groups were compared with shCtrl groups by the One-way ANOVA with the Tukey post-test. Data are expressed as mean ± SD (n = 3). *, p <0.05; **, p < 0.01; * *, p < 0.001.

We created an orthotopic pancreatic cancer nude mouse model to explore further the effects of pancreatic cancer on islet cells *in vivo*. In an effort to identify the effect of shTG2+shp53 pancreatic cancer cells on pancreatic β cells *in vivo*, we evaluated glucose and insulin tolerance *in vivo*. In the shTG2+shp53 and shTG2 groups, the tumor luminescence was significantly stronger compared to the other two groups (Figure 6A). Furthermore, the weight of the nude mice in all the groups was measured (Supplementary Figure 6A). The results demonstrated that glucose tolerance was reduced in the shTG2+shp53-treated pancreatic cancer mouse group during the 4^th^ to 6^th^ week (Figure 6CE). Conversely, glucose tolerance of the shTG2 group was eventually reduced without initial changes. In the shTG2-treated group, the glucose tolerance became reduced in the 5^th^ week in the BXPC3 cell and reduced in the 6^th^ week in the PANC1 cells. The glucose tolerance did not differ between the shp53-treated and the control groups (P >0.05) (Figure 6C–E). Furthermore, we did not observe any changes in insulin tolerance in any of the groups (Supplementary Figure 6B–D). These results suggest that silenced TG2 may be the cause of changes in the pancreatic cancer cells affecting β cells *in vivo* and that silenced p53 could exacerbate this phenomenon.

At the endpoint, the mice were euthanized and the tumors were weighed. Gross tumor morphology is shown in Figure 6B. The mean tumor weight of the shTG2 groups was significantly lighter compared to the other groups (Figure 6F). The tumor weight did not differ between the shTG2+shp53, shp53 and control groups (Figure 6F). The gross morphology and weight of the orthotopic pancreas and tumor are shown as Supplementary Figure 6E and F. Therefore, the relationship that was uncovered between TG2 and p53 indicates a possible mechanism for the development of hyperglycemia-associated pancreatic cancer.

## DISCUSSION

Hyperglycemia may be the first clinical manifestation of pancreatic cancer. [7, 9] However, the bidirectional interaction between pancreatic cancer and damage of βcell remains unclear[5, 6]. In this study, we attempted to investigate the mechanism by which glucose tolerance abnormality develops in pancreatic cancer dependent on microenvironmental stress. Our results demonstrated that silenced TG2 combined with p53 in pancreatic cancer cells may cause a specific microenvironment that decreases cell survival in pancreatic β cells *in vitro* and reduces glucose tolerance *in vivo*. Our findings establish roles for TG2 and p53 in the development of glucose tolerance abnormality-associated pancreatic cancer.

TG2 is ubiquitously expressed in cancer and is implicated in regulating several physiological processes[30]. Interestingly, p53 combined with TG2 might be involved in cellular regulation in response to metabolic stress resistance[31-33]. First, we showed that four pancreatic cell lines with various TG2 and p53 expressions demonstrated different sensitivities to glucose starvation. Our results demonstrated that TG2 protein level and activity were increased in glucose starvation. TG2 inhibition or shRNA treatment abolished resistance. Interestingly, resistance remained after combined targeted knockdown or inhibition of p53 in the pancreatic cells. Although p53 inhibition or shRNA treatment could also abolish resistance in glucose starvation, it abolished the sensitization of TG2 knockdown in glucose starvation. To investigate direct regulation by p53, our results clarified that nuclear p53 binds to TG2 at the transcription start site in glucose starvation stress by ChIP. Moreover, we clarified that resistance to glucose stress in shTG2+shp53 cells was attributed to induction of oxidative stress. Our results demonstrated that increased ROS production was acquired in control, shp53, and shTG2 cells under glucose starvation stress. Moreover, the higher ROS production was acquired in shTG2+shp53 and shTG2 cells when compared to control and shp53 cells. Therefore, control, shp53, and shTG2 cells underwent glucose starvation stress after treatment with NAC. Surprisingly, ROS inhibition did not significantly influence cell survival in shTG2+shp53 cells in response to glucose starvation stress. Furthermore, our results demonstrated that Bcl-2 expression was increased under glucose starvation stress compared to basal conditions. Therefore, the mechanism by which shTG2+shp53 cells remain resistant to glucose starvation may be explained as follows. ROS production is required for p53- and TG2-induced resistance under glucose starvation stress; however, increased ROS production is not the cause for resistance to glucose starvation stress in shTG2+shp53 cells.

In this context, we then analyzed whether silenced TG2 and p53 combination in pancreatic cancer cells affect adjacent pancreatic β cells. We found that the supernatant of shTG2+shp53 pancreatic cancer cells decreased cell survival by approximately 30% in pancreatic β cells after 72 h and 96 h of treatment. Inhibition of the ROS pathway played a protective role in β cells, while ROS higher production in the shTG2+shp53 pancreatic cancer cells may be responsible for damage in β cells. Microarray experiments demonstrated that gene expression in the MAPK and Cell cycle pathways was significantly affected. We assumed that the silenced TG2 and p53 combination provided a specific microenvironment affecting adjacent cells. This hypothesis was demonstrated in two ways: 1. shTG2+shp53 cells remained resistant to glucose starvation stress; 2. the highest ROS production was acquired in shTG2+shp53 cells. Through *in vivo* assay, we evaluated glucose and insulin tolerance in an orthotopic mouse model. The results demonstrated that glucose tolerance was reduced in the shTG2+shp53-treated group during the 4th to 6th week. In the shTG2-treated group, although glucose tolerance was unchanged in the 4th week, it became reduced in the 6th week. Furthermore, we did not observe any changes in insulin tolerance in any of the groups. Taken together, these results suggest that silenced TG2 may be the cause of changes in pancreatic cancer cells affecting β cells *in vivo* and that silenced p53 could exacerbate this phenomenon.

Our study differs from previous work focusing on possible mechanisms of glucose tolerance abnormality in pancreatic cancer. We revealed a possible mechanism by which pancreatic cancer impacts β cells through microenvironmental changes. Inhibition of TG2 and p53 increased intracellular ROS in pancreatic cancer cells. The supernatant of pancreatic cancer cells with TG2 and p53 combined interference decreased cell survival in pancreatic β cells. Glucose tolerance was abnormal for the pancreatic cancer mouse model with TG2 and p53 combined interference. Therefore, the uncovered relationship between TG2 and p53 proposes a possible mechanism by which glucose tolerance abnormality-associated pancreatic cancer may develop and could have therapeutic potential for cancer treatment and diagnosis.

In conclusion, we clarified that the sensitization effects of TG2 and p53 in glucose stress were attributed to induction of oxidative stress. Our results showed that the supernatant of pancreatic cancer cells with TG2 and p53 combined interference decreased cell survival in pancreatic β cells *in vitro*. Of note, our study demonstrated glucose tolerance abnormalities *in vivo* with TG2 and p53 combined interference, indicating a possible mechanism for hyperglycemia in pancreatic cancer. However, the mechanism for glucose tolerance abnormalities caused by pancreatic cancer with TG2 and p53 combined interference is also important and warrants further investigation.

## MATERIALS AND METHODS

### Materials and reagents

Cell culture medium, RPMI 1640, DMEM, trypsin-EDTA, and fetal bovine serum (FBS) were purchased from Gibco. The antibodies to actin, Histone H3, p53, TG2, Bcl-2, horseradish peroxidase–conjugated goat anti-rabbit, and sheep anti-mouse were purchased from Cell Signaling Technology. The p53 inhibitor (Pifithrin-α) was purchased from Sigma Aldrich, TG2 inhibitor (cystamine dihydrochloride) was purchased from R&D, Bcl-2 inhibitor (ABT-737) was purchased from Selleckchem, 5-(Biotinamido)pentylamine and FITC-conjugated streptavidin were purchased from Thermo Fisher Scientific, and N-acetyl-L-cysteine(NAC) and dihydroethidium were purchased from Beyotime Biotechnology. All organic reagents used in this study were purchased from Sinopharm (Shanghai, China).

### Cell lines and cultures

The human pancreatic cancer cell lines BXPC3, PANC1, ASPC1, and SW1990 were purchased from the cell bank of the Chinese Academy of Science (Shanghai, China). The BXPC3 and PANC1 cells were stably transfected with the luciferase gene using pcDNA3.1 vector maintained in 50 ug/ml puromycin dihydrochloride. The rat pancreatic islet β-cell min6 were purchased from Shanghai Biological Technology. All of the cell lines (except ASPC1 in RPMI 1640) were cultured in DMEM supplemented with 10% FBS, 100 μg/ml penicillin, and 100 μg/ml streptomycin in a humidified incubator in 5% CO_2_ at 37°C.

### In situ TG2 enzymatic activity assay

In situ TG2 enzymatic activity was measured using a pseudosubstrate of TG2 according to the published protocol[30]. In brief, cells were incubated with 5 mM 5-(biotinamido)pentylamine (BAP; Thermo Fisher Scientific) for 2 h at 37°C. The cells were fixed with 4% formaldehyde for 30 min. Following blocking with 5% BSA for 60 min, the cells were incubated with FITC-conjugated streptavidin (1: 200) (Thermo Fisher Scientific) for 1 h and observed under confocal microscopy. TG2 activity was detected using a microscope (Leica TCS SP5).

### TG2 and p53 adenovirus transfection

The specific antihuman TG2 shRNA sequences (5′-GGCCCGTTTTCCACTAAGA-3′) and antihuman p53 shRNA sequences (5′-GACUCCAGUGGUAAUC UAC(TT)-3′) were constructed into adenovirus using GV248vector. The adenovirus containing the TG2 and p53 cDNA construct was provided by Shanghai Gene chem. As a negative control, a non-targeting shRNA vector was used. For lentiviral infection, BXPC3 and PANC1 cells were grown in six-well culture plates with a density of 5 × 10^5^ cells per well overnight. According to the manufacturer’s protocol, the cells were incubated with polybrene (50 μg/ml) with the addition of the lentiviral vector (approximately 100 mM of infection) for 6 h. After being replaced in refreshed medium, the stable transfectedcell lines were selected in 50 μg/ml puromycin dihydrochloride and maintained in growth medium containing 10 μg/ml puromycin dihydrochloride.

### Western blot analysis

Nuclear extracts and whole-cell extracts were obtained as provided by the Beyotime Biotechnology with RIPA and nuclear extract buffer containing 1 mM PMSF. In brief, extracted protein (40 μg) was subjected to SDS-PAGE and transferred onto polyvinylidene fluoride (PVDF) membranes. The mouse anti-human TG2 mAb (final dilution 1:1000), the rabbit anti-human P53 mAb (final dilution 1:1000), or the rabbit anti-human Bcl-2 mAb (final dilution 1:1000) were used as the primary antibody and the HRP–conjugated goat anti-rabbit (final dilution 1:1000) or sheep anti-mouse IgG (final dilution 1:1000) was used as the secondary antibody. The bands were detected with the Enhanced Chemiluminiscence (ECL) kit (GE Healthcare) and visualized with the G Box Chemic XL system (Gene Company Limited). Quantitative analysis of protein expression was performed by normalizing against actin or His3A with the image J.

### Reactive oxygen species measurement

For quantification of intracellular ROS levels, cells were loaded with 5 μM dihydroethidium (Beyotime Biotechnology) for 30 min at 37°C and 5% CO_2_. Cells were collected and washed twice with PBS and suspended in 500 μL PBS. Mean fluorescence intensity was used as a measure of ROS as determined by flow cytometry FACSCalibur (BD Biosciences) using CellQuest Pro 5.2 with an excitation wavelength of 488 nm and an emission wavelength of 610 nm.

### Chromatin immunoprecipitation (ChIP)

To examine endogenous binding of p53 to TG2, BXPC3 and Panc1 cells were treated with glucose deprivation for 2 days. ChIP was performed using the EZ ChIPTM Kit (Millipore) according to the manufacturer’s instructions. In brief, cells were fixed using 4% formaldehyde and treated with lysis buffer. After sonification, specific protein–DNA complexes were immunoprecipitated using anti-p53 antibody (Santa Cruz Biotechnology) with IgG as controls. Using specific primers (Table 1) upstream of the promoter TG2 transcription start site, qPCR was then performed. TG2 was calculated and the background signal was subtracted. F1R1(300-500bp), F2R2(700-900bp) F3R3(2000-2200bp), F4R4(3600-3800bp), F5R5(5000-5200bp), and F6R6(5900-6100bp) were demonstrated as shown in Table 1.

**Table 1:** The sequence of primers for TG2 response elements

Primers	Forward primer (5’-3’)	Reverse primer (5’-3’)
300-500bp	GGAAGGGATGAATGTGGAAGAT	GTGAGAATCACATCCCAGACC
700-900bp	CACCTGCCTCCTGACATTTTC	AGGGAGCATAGGGAGGTG
2000-2200bp	TGAGGTAAGACAGGGCACAG	TGGATTCCAGTCCCAGTGAC
3600-3800bp	GGTTTGTTCACTGAGACTCCAG	TGACATATGGCTCAGGGAAAAC
5000-5200bp	CGGGACTGGGCAATGGGTG	GAGAGCGGCGCTAACTTATAG
5900-6100bp	TGCCAACTCTGGCTTCCAGG	CACCTGCCCAGTGTCAGCA

### Cell viability assays

Cell viability assays were measured using the Cell Counting Kit-8 kit (CCK-8, Dojindo laboratories, Kumamoto, Japan). In brief, cells were seeded in 96-well plates (5×10^3^ cells per well) overnight. Cells pretreated with inhibitors (pifithrin-α or cystamine dihydrochloride) or transfected as described above with shRNA (control, TG2 or p53) were incubated with a series of concentrations of glucose culture medium. The cells were pretreated with NAC or ABT. After 48 h or 72 h, cell viability was evaluated by adding 10 μl of CCK-8 solution to each well of the plate. According to the manufacturer’s standard protocol, cell viability was measured using a BIO-TEK ELx800 Universal Microplate Reader (Bio-Tek, Winooski, VT, USA) by determining the absorbance (450 nm) after incubation for 2 h at 37°C. Cell viability was calculated using the formula: [(AE – AB)/(AC – AB)] × 100%. AE, AC, and AB were defined as the absorbance of experimental samples, untreated samples, and blank controls, respectively.

### Animal studies

All mice were purchased from the Shanghai Experimental Animal Center of the Chinese Academy of Sciences (Shanghai, China). The mice were placed in a pathogen-free environment and allowed to acclimatize for a week before being used in the studies. BXPC3 (control, shTG2, shp53, shp53+shTG2) cells or PANC1 (control, shTG2, shp53, shp53+shTG2) cells (3*10^6^) were injected into the pancreatic subcapsule of the 5-week-old nu/nu (nude) mice. The successful establishment of an orthotopic PanCa model was demonstrated by IVIS Lumina Imaging System (Perkin Elmer), which was taken to capture visible light photographic and luminescent images. The mice were monitored for body weight at three-day intervals. Starting in the 4^th^ week, glucose and insulin tolerance tests were performed on the mice. Glucose tolerance test: After overnight fasting, mice were subjected to an intraperitoneal injection of glucose (2 mg/g body weight) followed by blood glucose measurement at multiple time points. Blood samples were obtained from the tail vein at 0, 15, 30, 60, and 120 min after glucose load. Blood glucose levels were measured with a glucose analyzer (OneTouch Ultra, Johnson & Johnson, Milpitas, CA, USA). Insulin tolerance test: After overnight fasting, mice were subjected to an intraperitoneal injection of insulin (0.5 mU/kg body weight) followed by blood glucose measurement at multiple time points with a glucose analyzer (OneTouch Ultra, Johnson & Johnson). On the 7^th^ week, the mice were euthanized and the tumors were excised and weighed.

### Statistical analysis

Data in this study were analyzed and graphed by the GraphPad Prism statistics program. A direct comparison between two groups was performed using Student’s t test, and the results of three or more groups were performed using one-way ANOVA with the Tukey post-test. P value of < 0.05 was considered statistically significant. *, P < 0.05; **, P < 0.01; ***, P < 0.001; ns represents not significant (P > 0.05).

## SUPPLEMENTARY MATERIALS FIGURES



## Abbreviations

TG2: tissue transglutaminase2
PanCa: pancreatic ductal adenocarcinoma
