# Supplementary material for: Effect of p53 on pancreatic cancer-glucose tolerance abnormalities by regulating transglutaminase 2 in resistance to glucose metabolic stress
Source: Oncotarget. 2017 Jul 19;8(43):74299–311. doi: 10.18632/oncotarget.19402 (PMC5650341; doi:10.18632/oncotarget.19402)
Supplement: Supplementary file 1 [file oncotarget-08-74299-s001.pdf]

Effect of p53 on pancreatic cancer-glucose tolerance abnormalities by regulating transglutaminase 2 in resistance to glucose metabolic stress

SUPPLEMENTARY MATERIALS

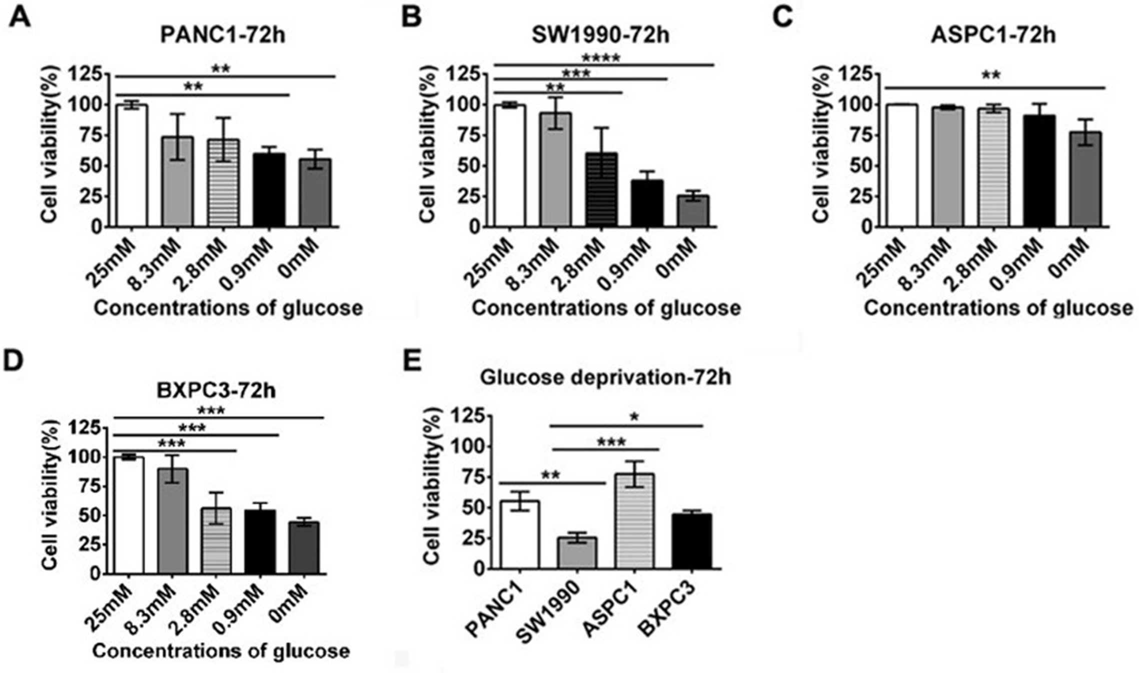

Supplementary Figure 1: (A-D) The cell viability of PANC1, ASPC1, SW1990, BXPC3 cells treated with a series concentrations of glucose deprived cultural medium for 72 h. **(E)** The differential cell viability to glucose starvation in the four cell lines at 72h. Data are expressed as mean ± SD (n = 3). *, p <0.05; **, p < 0.01; * *, p < 0.001.

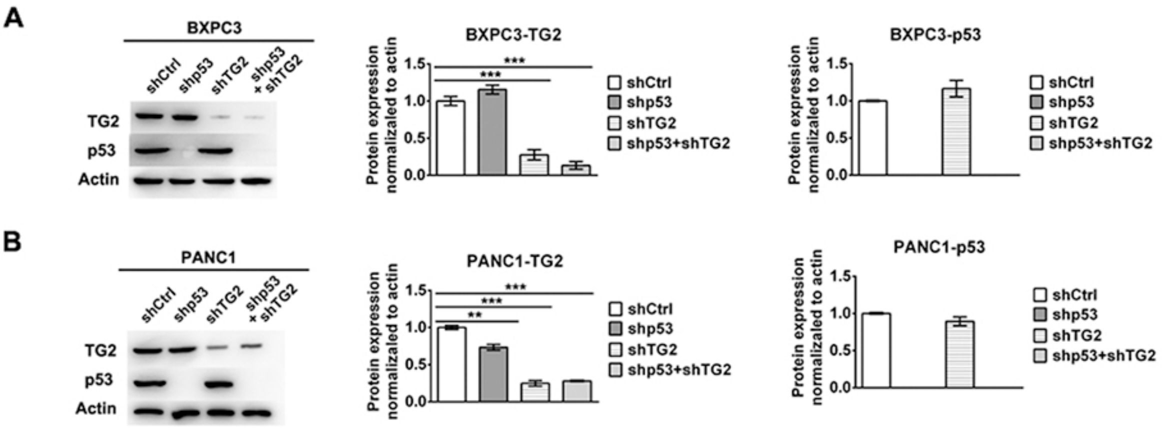

Supplementary Figure 2: Western blot analysis of the expression of TG2 and p53 in pancreatic cancer cells BXPC3 **(A)** and PANC1 **(B)** after shRNA transfection. The protein expression normalized to actin was analyzed. Data are expressed as mean ± SD (n = 3). *, p <0.05; **, p < 0.01; * *, p < 0.001.

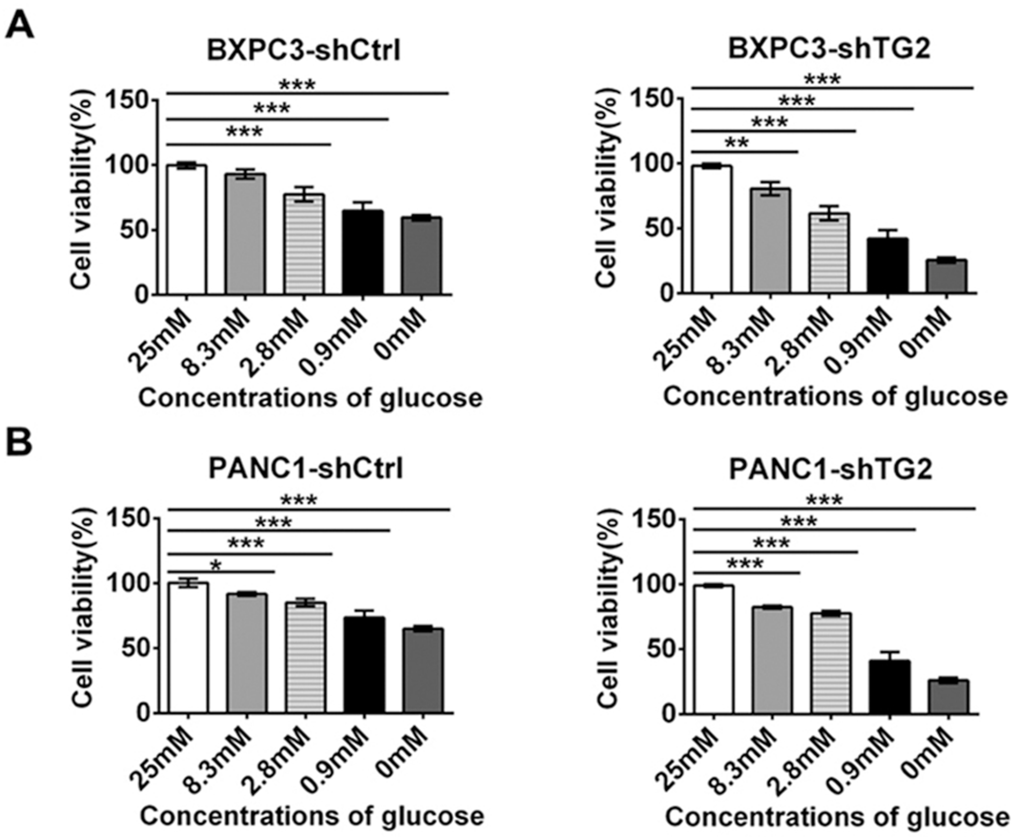

Supplementary Figure 3: The cell viability of BXPC3 **(A)** and PANC1 **(B)** cells with shCtrl or shTG2 transfection in a series concentrations of glucose deprived cultural medium for 48h. Data are expressed as mean ± SD (n = 3). *, $p < 0.05$; **, $p < 0.01$; **, $p < 0.001$.

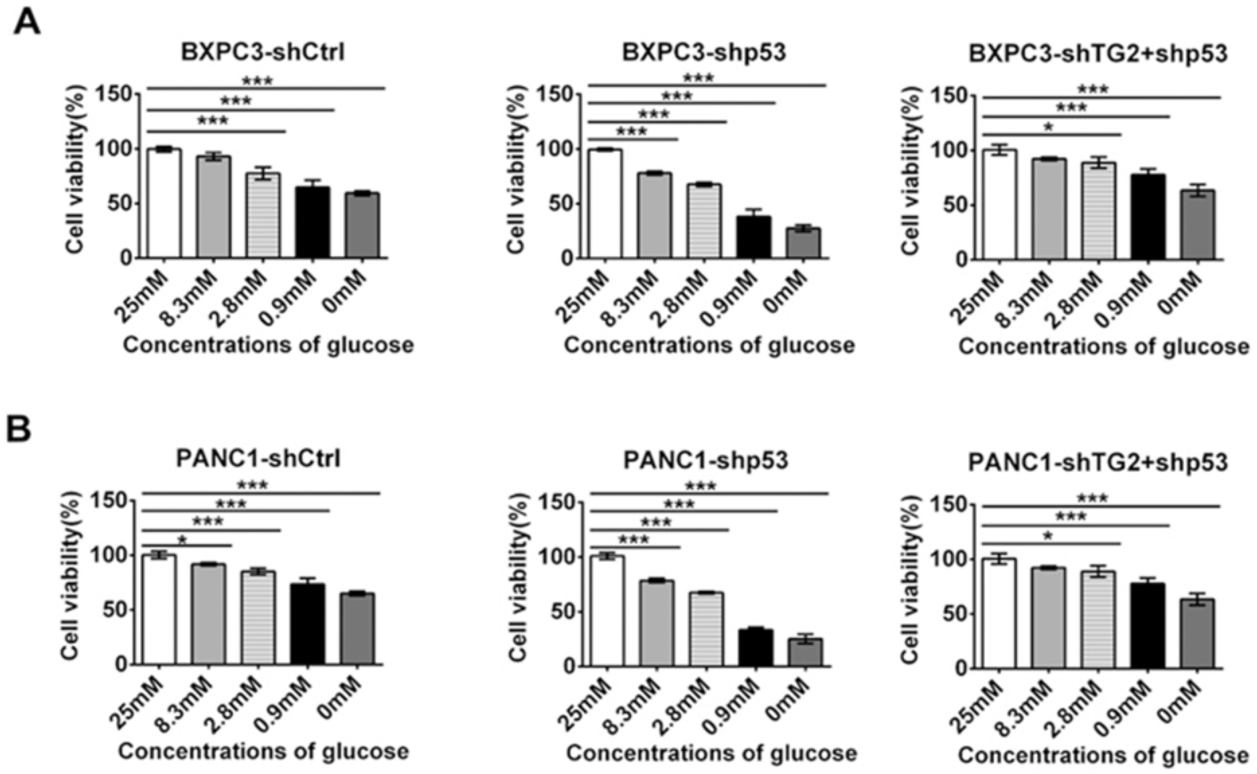

Supplementary Figure 4: The cell viability of BXPC3 **(A)** and PANC1 **(B)** cells with shCtrl, shp53 or shTG2+shp53 transfection in a series concentrations of glucose deprived cultural medium for 48h. Data are expressed as mean ± SD (n = 3). *, p <0.05; **, p < 0.01; * *, p < 0.001.

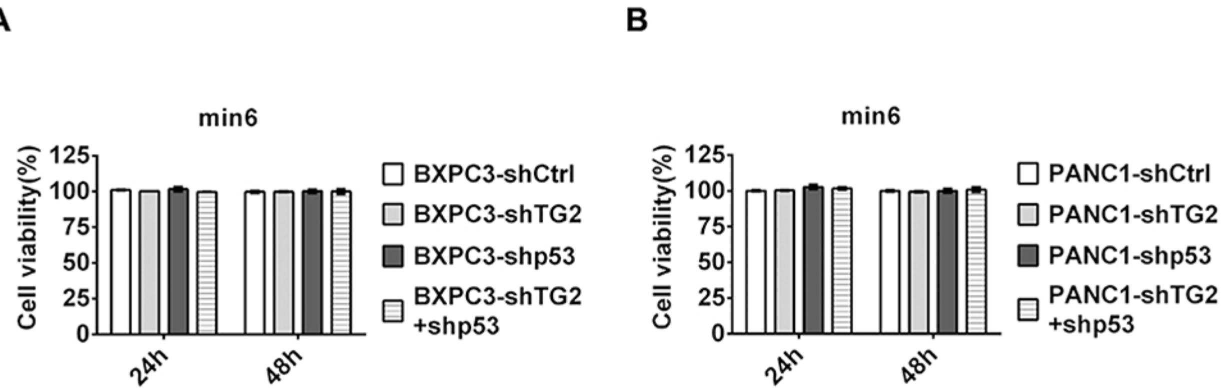

Supplementary Figure 5: The supernatant of BXPC3 **(A)** and PANC1 **(B)** cells expressing shCtrl, shTG2, shp53 or shTG2+shp53 treated pancreatic β cell min6. The cell survival in min6 were measured after 24h, 48h treatment. Data are expressed as mean ± SD (n = 3). *, p <0.05; **, p < 0.01; * *, p < 0.001.

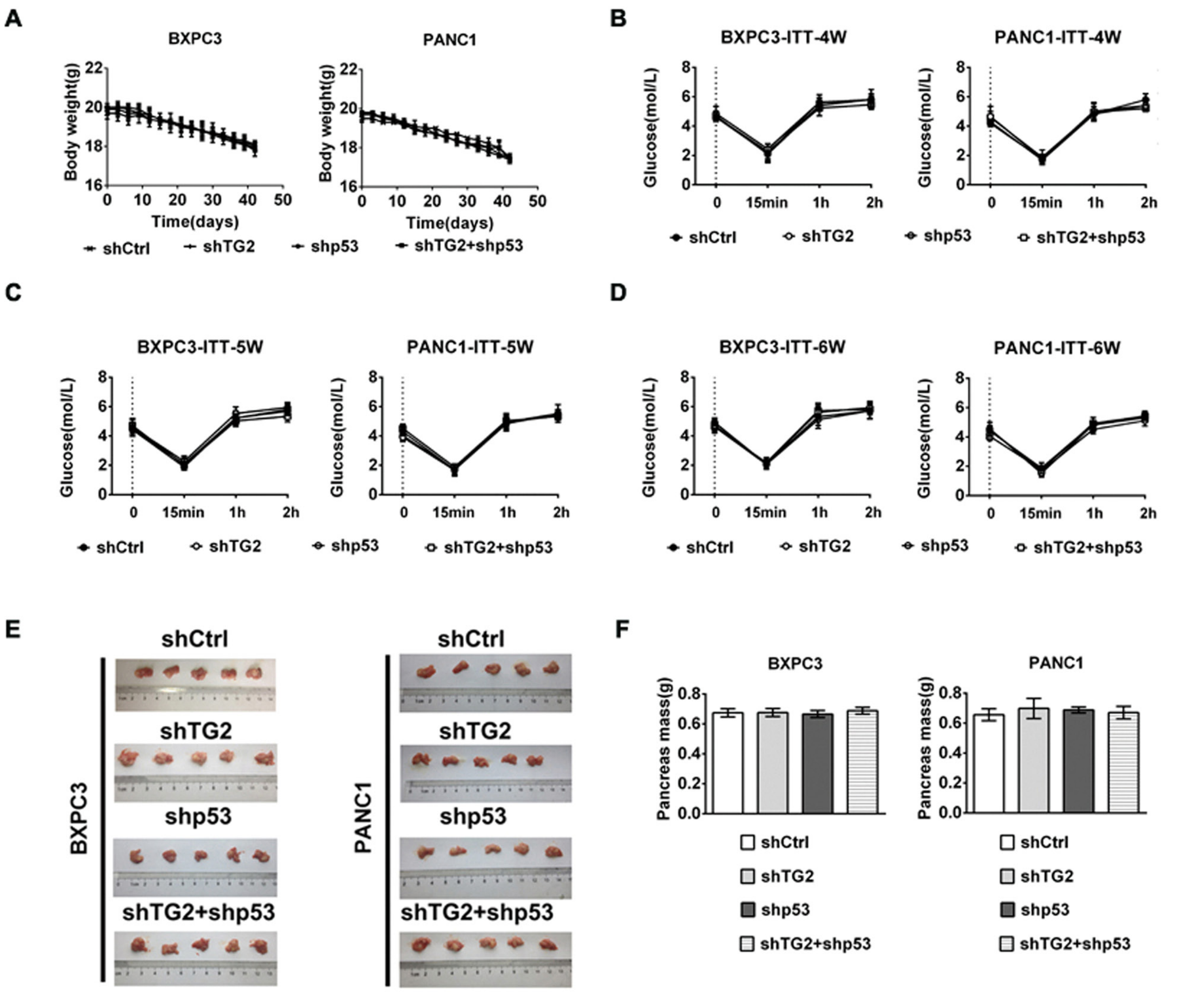

Supplementary Figure 6: The orthotopic pancreatic mice model of 4 groups, shCtrl, shTG2, shp53 or shTG2+shp53 were successfully established for 5 mice per group. **(A)**The weight change of the mice during the treatment. **(B) (C) (D)** Insulin tolerance test in the 4 groups mice at 4th, 5th, 6th week. **(E)** Gross morphology of orthotopic pancrease and tumor. **(F)** The excised pancrease were weighted. The weight of the 4 groups were compared with shCtrl groups by the One-way ANOVA with the Tukey post-test. Data are expressed as mean ± SD (n = 3). *, p <0.05; **, p < 0.01; * *, p < 0.001.
